# Mechanistic investigation of SARS-CoV-2 main protease to accelerate design of covalent inhibitors

**DOI:** 10.1038/s41598-022-23570-6

**Published:** 2022-12-05

**Authors:** Hoshin Kim, Darin Hauner, Joseph A. Laureanti, Kruel Agustin, Simone Raugei, Neeraj Kumar

**Affiliations:** 1 Physical and Computational Science Directorate, Pacific Northwest National Laboratory, Richland, WA 99352 USA; 2 Earth and Biological Science Directorate, Pacific Northwest National Laboratory, Richland, WA 99352 USA

**Keywords:** Biochemistry, Enzyme mechanisms, Computational models

## Abstract

Targeted covalent inhibition represents one possible strategy to block the function of SARS-CoV-2 Main Protease (M^PRO^), an enzyme that plays a critical role in the replication of the novel SARS-CoV-2. Toward the design of covalent inhibitors, we built a covalent inhibitor dataset using deep learning models followed by high throughput virtual screening of these candidates against M^PRO^. Two top-ranking inhibitors were selected for mechanistic investigations—one with an activated ester warhead that has a piperazine core and the other with an acrylamide warhead. Specifically, we performed a detailed analysis of the free energetics of covalent inhibition by hybrid quantum mechanics/molecular mechanics simulations. Cleavage of a fragment of the non-structured protein (NSP) from the SARS-CoV-2 genome was also simulated for reference. Simulations show that both candidates form more stable enzyme-inhibitor (E-I) complexes than the chosen NSP. It was found that both the NSP fragment and the activated ester inhibitor react with CYS145 of M^PRO^ in a concerted manner, whereas the acrylamide inhibitor follows a stepwise mechanism. Most importantly, the reversible reaction and the subsequent hydrolysis reaction from E-I complexes are less probable when compared to the reactions with an NSP fragment, showing promise for these candidates to be the base for efficient M^PRO^ inhibitors.

## Introduction

The novel coronavirus pandemic (COVID-19) has prompted the need for new therapeutics to counter the threat of emerging viral pathogens. This pandemic has resulted in over 530 million infections and more than 6.3 million deaths worldwide, based on data from the World Health Organization (WHO) as of Jun. 2022^1^. The human cost has been further compounded by sweeping economic distress. Despite vaccines against the COVID-19 agent, the severe acute respiratory syndrome coronavirus 2 (SARS-CoV-2), has been deployed^2,3^, they are not 100% effective, and vulnerable populations, such as those with compromised immune systems or chemotherapy patients, cannot receive them. Consequently, the discovery of antiviral drugs targeting the virus by inhibiting various SARS-CoV-2 proteins, and their variants, is of immense importance for treating patients^4–6^. Two viral cysteine proteases, the main protease (M^PRO^) and the papain-like protease (PL^PRO^)^7^, have attracted research interest because of their role in viral replication. After the infection is initiated, viral genomic RNA is released into the cytoplasm and translated into replicase polyproteins, so-called pp1a or pp1ab; then M^PRO^ and PL^PRO^ proteolytic cleavage of these polyproteins results in the formations of non-structured proteins (NSP1-NSP16)^8,9^. These NSPs eventually form replicase-transcriptase complexes, which are implicated in the synthesis of a full-length genome, thus leading to viral replications^7^.

**Figure 1:**
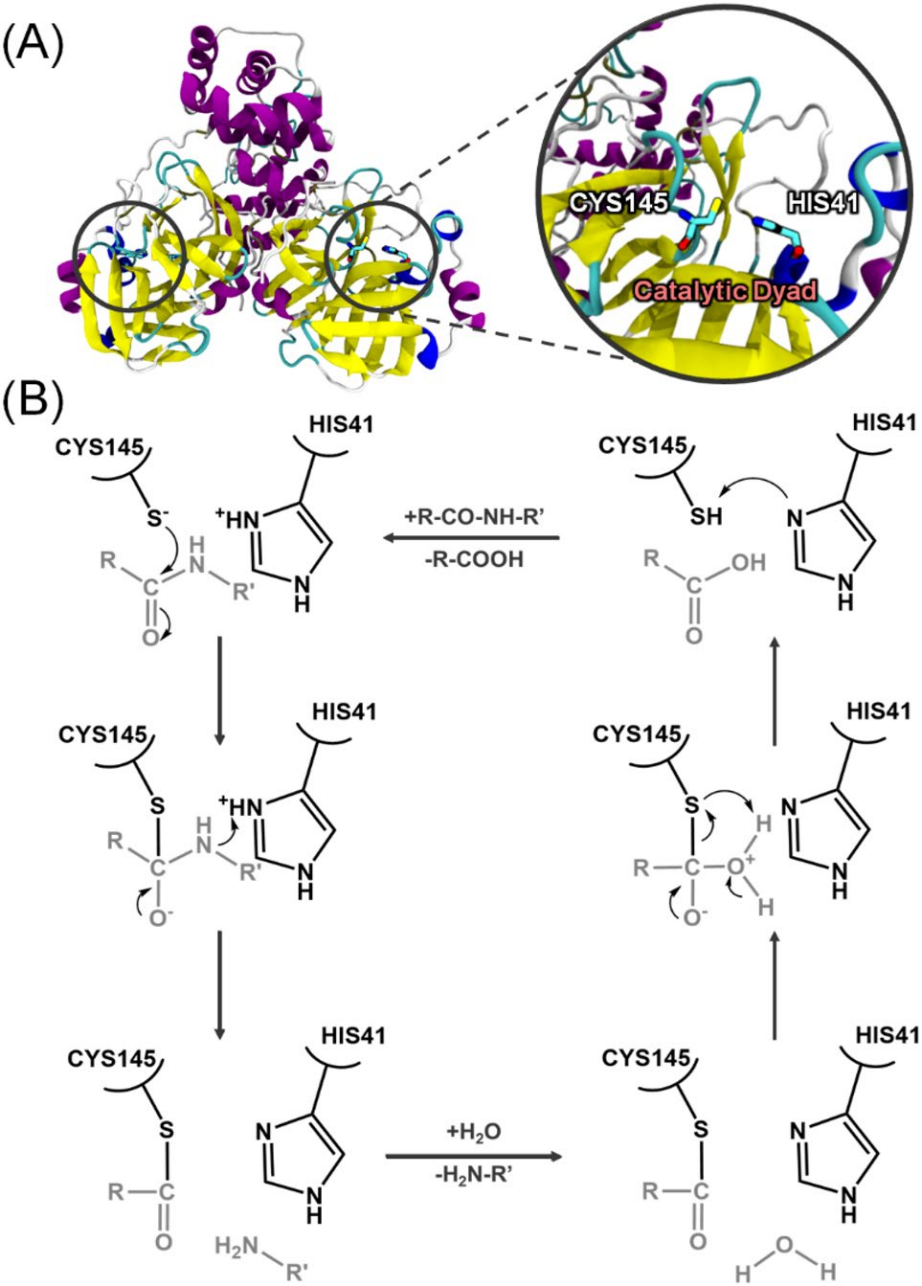
(**A**) Homo-dimeric crystal structure of M^PRO^ (PDB: 6WQF) with a highlight of the catalytic dyad, CYS145, and HIS41. Helices, beta-sheets, turns are colored by purple, yellow, and cyan, respectively. (**B**) Possible reaction mechanism of cysteine proteases.

The present work focuses on the covalent inhibition of M^PRO^, as recent high-resolution crystal structures of SARS-CoV-2 M^PRO^ provide a template for developing non-covalent and covalent inhibitors^10,11^. M^PRO^ is a homodimer, which features a cleft near the surface of each monomer, containing a catalytic dyad composed of a cysteine (CYS145, numbering according to protein data bank entry 6WQF) and histidine (HIS41) (Fig. 1A). Recent studies highlighted that ligand binding to the M^PRO^ can induce significant structural changes at the active site. For example, large structural flexibility was observed in a small helix region (residues 46–50) and a loop region (residues 190–195) upon ligand binding^11^. Furthermore, it has been suggested that the binding of small molecules to one subunit influences the structure of the other subunit^12^.

According to the commonly accepted general mechanism of cysteine proteases, M^PRO^ catalysis starts with the deprotonation of the thiol group of CYS145 by HIS41 (Fig. 1B). The next step is the nucleophilic attack of the thiolate anion to the carbonyl group of the peptide bond to be cleaved. Protonation of the nitrogen atom by HIS41 leads to the release of a fragment of product with an amine end. The nucleophilic attack might also proceed in a concerted manner with the simultaneous formation of a sulfur–carbon bond and the breaking of the substrate carbon–nitrogen bond, thus forming a thioester intermediate. The thioester is then hydrolyzed by water, resulting in the product release and the reaction starts over.

An extensive effort has been made to develop antiviral non-covalent inhibitors against M^PRO^ via high throughput virtual screening (HTVS) using a docking workflow^13,14^ followed by experimental validation^10,11,15^. However, the design of covalent inhibitors has been less common for the SARS-CoV-2 M^PRO^ due to the lack of mechanistic details and electrophilic warhead design strategies^16^. This is somehow surprising as targeted covalent inhibitors represent a viable strategy to inhibit M^PRO^ involved in different pathologies, not just SARS-CoV-2.

Ideally, a covalent M^PRO^ inhibitor should mimic the recognition motif of non-structured peptides (NSPs) and bear a reactive electrophilic group (‘warhead’) to covalently bind with CYS145 of M^PRO^ yielding a stable enzyme-inhibitor (E-I) complex. In general, a surge has been observed recently in inhibitor design with various warheads that efficiently inhibit enzymatic reactions. Libraries of such inhibitors include acrylamides, ester, chloroacetaamides, nitriles, disulfides, maleimides, ketones, and pyrodines, and may be generated using deep learning models, such as 3D-Scaffold^17^.

Despite recent efforts to computational design for covalent inhibitors against SARS-CoV-2 protein targets^18^, a little effort has been dedicated to elucidating the reaction inhibition mechanism at an atomistic level^19^. We contend that to design potent covalent inhibitors we need to obtain a detailed atomistic understanding of both the catalytic mechanism of M^PRO^ and the potential inhibition pathways, which in turn can be used to inform the design of new targets with improved inhibitory properties. In this context, atomistic simulations have been carried out to investigate the catalytic mechanisms taking fully into account the network of interactions at the active site^19–21^. For example, quantum mechanics/molecular mechanics (QM/MM) molecular dynamics (MD) simulations were used to explore the free energetics of inhibition by Michael acceptors and showed that these molecules form stable E-I complexes with M^PRO^.^22^ Another QM/MM study showed that when a peptidyl Michael acceptor is used the E-I complex is formed in a concerted manner rather than a stepwise manner^22^. More recently, multiscale QM/MM MD simulations were employed to explore the reaction mechanisms of natural substrates and various inhibitors of both M^PRO^ and PL^PRO^^23–27^.

We employed an in-house covalent inhibitor docking workflow to select candidates from the warhead library containing thousands of potential covalent inhibitors based on acrylamide and ester warheads. Then, using QM/MM simulations, we investigated the mechanism of hydrolysis of NSPs and the inhibition by two selected activated esters and acrylamide warheads. Results are then compared to infer general principles to inform the design of new inhibitors.

## Results and discussions

### High throughput covalent docking simulations

**Figure 2:**
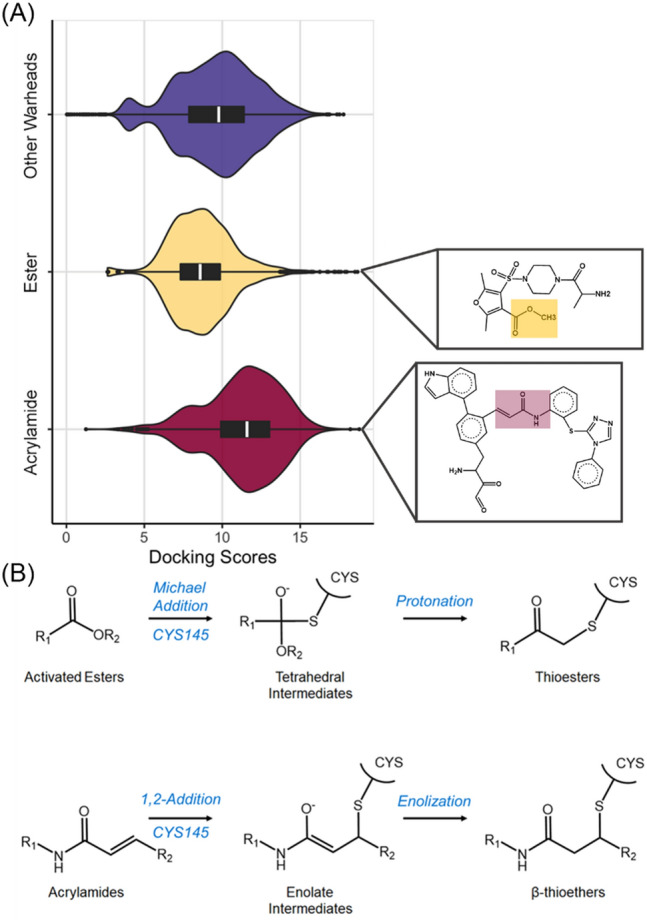
(**A**) Violin plots of the distribution of the docking score against the M^PRO^ target of the ester and acrylamide warheads, and those from other warheads (chloroacetaamides, nitriles, disulfides, maleimides, and pyrodines), whereby higher values imply more favorable covalent binding. (**B**) the proposed electrophilic addition to CYS145 is shown for the acrylamide and ester warhead selected for used in our covalent workflow.

We performed a high throughput virtual screening against M^PRO^ using a covalent docking workflow developed by our group. The workflow was applied to our curated covalent antiviral datasets^17^ built from multiple sources^28,29^. This dataset was used to train a 3D-Scaffold deep learning model to generate new antiviral candidates^17^. The dataset comprises candidates from FDA-approved drugs and candidates from the enamine database with various functional groups (so-called scaffold), including acrylamides, ester, chloroacetaamides, nitriles, disulfides, maleimides, and pyrodines. In particular, acrylamide^30^ and ester warheads^31^ have been suggested to be potent protease inhibitors. Consistently, our covalent screening indicated that these warheads are indeed the most promising covalent candidates (Fig. 2), as detailed below. For this reason, in this work, we focused on two of the top-ranking acrylamide and ester warheads (Fig. 2, insets).

**Figure 3:**
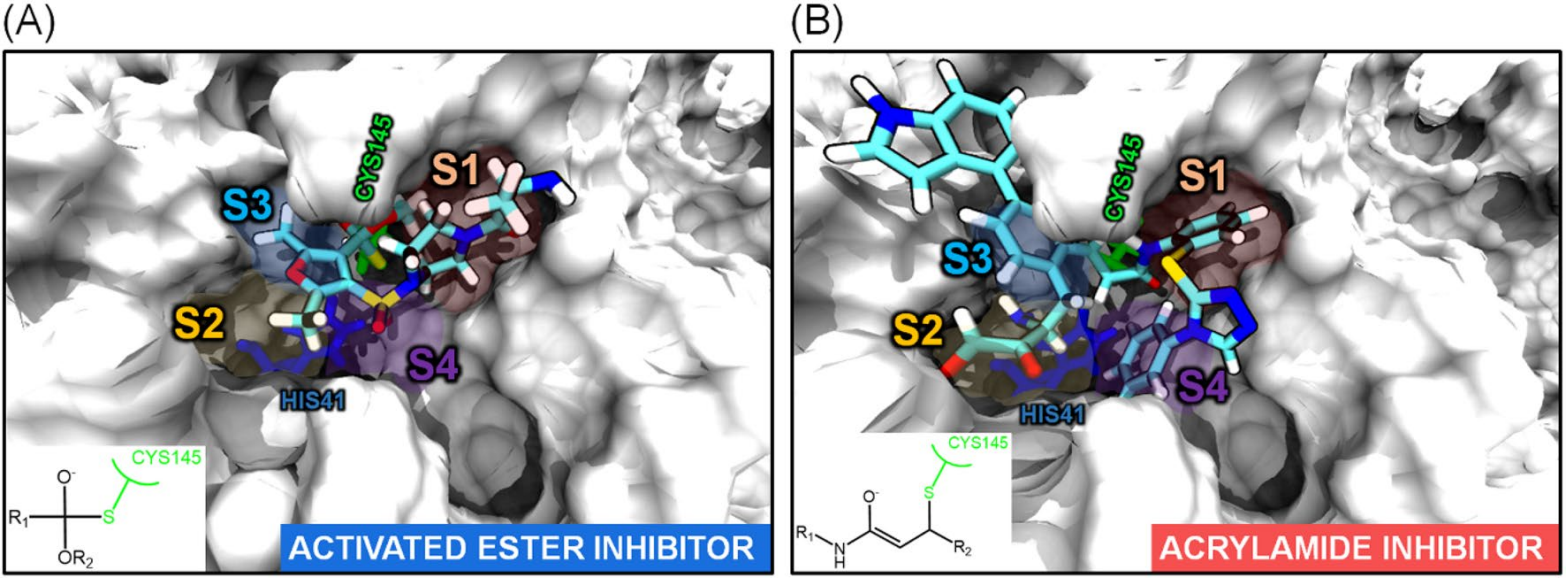
Observed binding modes of covalently bound (**A**) ester and (**B**) acrylamide warheads following electrophilic attack at CYS145 of M^PRO^. Blue, cyan, red, yellow, and white licorice representations denote nitrogen, carbon, oxygen, sulfur, and hydrogen atoms respectively. S1–S4 binding pockets are represented by orange, yellow, blue, and purple shadows, respectively.

We screened a library of 5000 acrylamide and ester warheads, targeted towards cysteine residues, using specific functional moieties^17^ that can react irreversibly with CYS145 of the M^PRO^. These functional groups exhibit strong interactions with the nucleophilic thiol (SH) group of CYS145. A virtual high-throughput covalent screening based on a flexible docking framework was performed to rank the binding affinity of the ligands in the library to CYS145 (Fig. 2). This step involves the covalent bond formation and binding pose prediction (see “Method” section). We identified the top candidates as those with the highest binding score and favorable interactions within the binding pockets S1, S2, S3, and S4 of M^PRO^ as obtained by direct visual inspection (Fig. 3). We finalized two top-ranked candidates that were subsequently used for mechanistic studies (Fig. 2, insets): methyl 4-((4-alanylpiperazin-1-yl)sulfonyl)-2,5-dimethylfuran-3-carboxylate and 2) 3-(5-(2-amino-3,4-dioxobutyl)-2-(1H-indol-4-yl)phenyl)-N-(2-((5-phenylcyclopenta-1,3-dien-1-yl)thio)phenyl)propenamide. Hereafter, these two candidates are indicated as the activated ester (AE) inhibitor and the acrylamide (AA) inhibitor, respectively.

We evaluated physiochemical properties, Lipinski’s Rule of Five, and Absorption, Distribution, Metabolism, and Excretion (ADME) parameters for both candidates using SwissADME^32^. These candidates are predicted to permeate the blood–brain barrier (synthetic accessibility score which is below 5) and have both a bioavailability of 0.55.

### Molecular dynamics simulations of M^PRO^/ligand covalent complexes

We performed MD simulations of these two candidates and an additional non-structural protein (NSP) fragment for comparative analysis to understand how the presence of these three substrates influences the global dynamics of M^PRO^.

The overall structure of the ligand-free and substrate-bound M^PRO^ obtained from MD simulations does not deviate appreciably from the crystallographic structure (PDB ID: 6WQF)^11^. The root mean square deviations (RMSD) with respect to the crystal structure over a trajectory of 100 ns ranged from 2.0 to 2.5 Å for all cases (Fig. S1). All systems also show similar global dynamical features as indicated by the root mean square fluctuations (RMSF) of the individual amino acids (Fig. S1).

**Figure 4:**
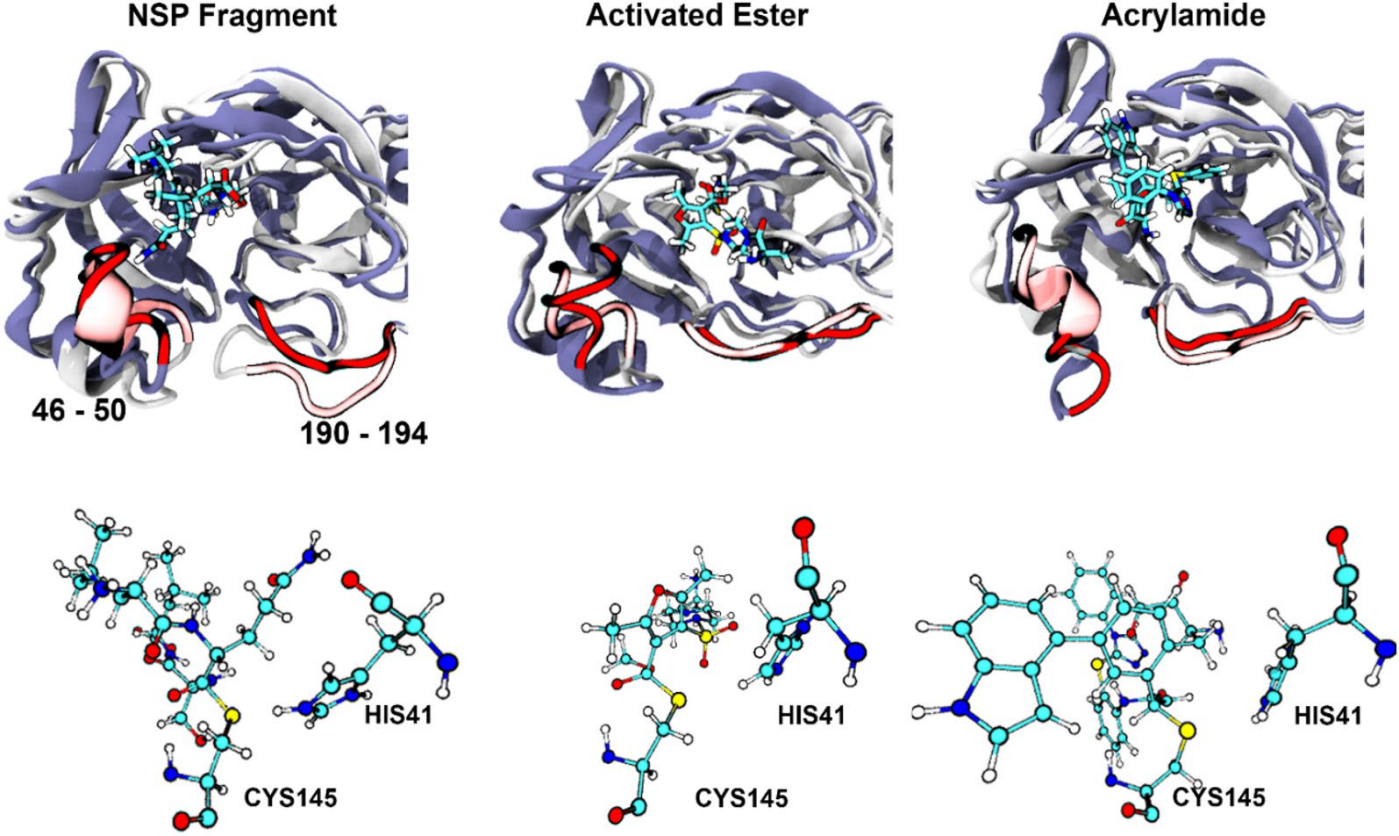
Active site structure of M^PRO^ with and without substrates (NSP, Activated Ester, Acrylamide). Top panels: superimposed images of the ligand-free (white cartoon model) and substrate-bound M^PRO^ active site (light blue cartoon model). Regions with high flexibility in two different states are highlighted as red (ligand-bound state) and light red (ligand-free state). Bottom panels: representative snapshots of the position of the catalytic dyad (HIS41 and CYS145) with respect to the substrates.

Recent studies have revealed that the presence of a ligand in the catalytic pocket of M^PRO^ can induce large structural flexibility around the catalytic pocket^11^ or even influence the tertiary structure of the other subunit of M^PRO^^12^. To explore the effect of substrate binding on the dynamics of either the active site residues or the entire subunit, we performed MD simulations where the truncated non-structured protein (NSP) 14–15 fragment (see below), and the AE and AA inhibitors were covalently bound to only one subunit while the other subunit was kept ligand-free. These simulations demonstrated that there are three flexible regions with relatively high RMSF values of the substrate-bound subunit with respect to the ligand-free subunit. These flexible regions include residue 46–54, 189–192 (located near the active site), and 275–279 (located at the periphery) and the differences between these three regions are observed in all cases we studied (Fig. S1). No change in the orientation and position of the catalytic dyad is observed upon ligand binding (Figs. 4 and S1).

### Cleavage of the NSP fragment

The catalytic mechanism of hydrolysis of the peptide bond of a naturally occurring M^PRO^ substrate and the potential mechanism of inhibition of the two selected warheads was investigated via hybrid QM/MM MD simulations.

We started by investigating the hydrolysis of a model of the NSP 14–15 fragment (LEU-TRP-ASN-THR-PHE-THR-ARG-**LEU-GLN-|-SER-LEU**-GLU-ASN-VAL-ALA-TYR-ASN-VAL, where the bold amino acids represent recognition sequence and the symbol ‘|’ represents the cleavage site). The first committed step in the cysteine proteases mechanism is the deprotonation of the catalytic cysteine thiol by an adjacent amino acid with a basic side chain, usually a histidine residue as in this case. Estimates of the p*K*_a_ values of CYS145 (p*K*_a_ = 7.1) and HIS41 (p*K*_a_ = 6.1) indicate that deprotonation of CYS145 is a thermodynamically facile step under physiological conditions. The estimated low p*K*_a_ values for both residues, which we recall are exposed to the aqueous environment, also suggest that they are likely to be ionized at neutral pH, and deprotonation of CYS145 by HIS41 might not be needed. Based on these considerations, all the QM/MM simulations were started from an ionized catalytic dyad.

A nucleophilic attack by the cysteinate anion to the carbonyl carbon of glutamine (GLN) in the NSP yields a tetrahedral intermediate, followed by deprotonation of histidine via proton transfer to the backbone amide of serine (SER) of NSP (Fig. 1B). The peptide bond between GLN and SER is cleaved and a product containing an amino terminus is released while the substrate with the carboxy terminus binds to the cysteine as a form of thioester (Figs. 1 and 4).

**Figure 5:**
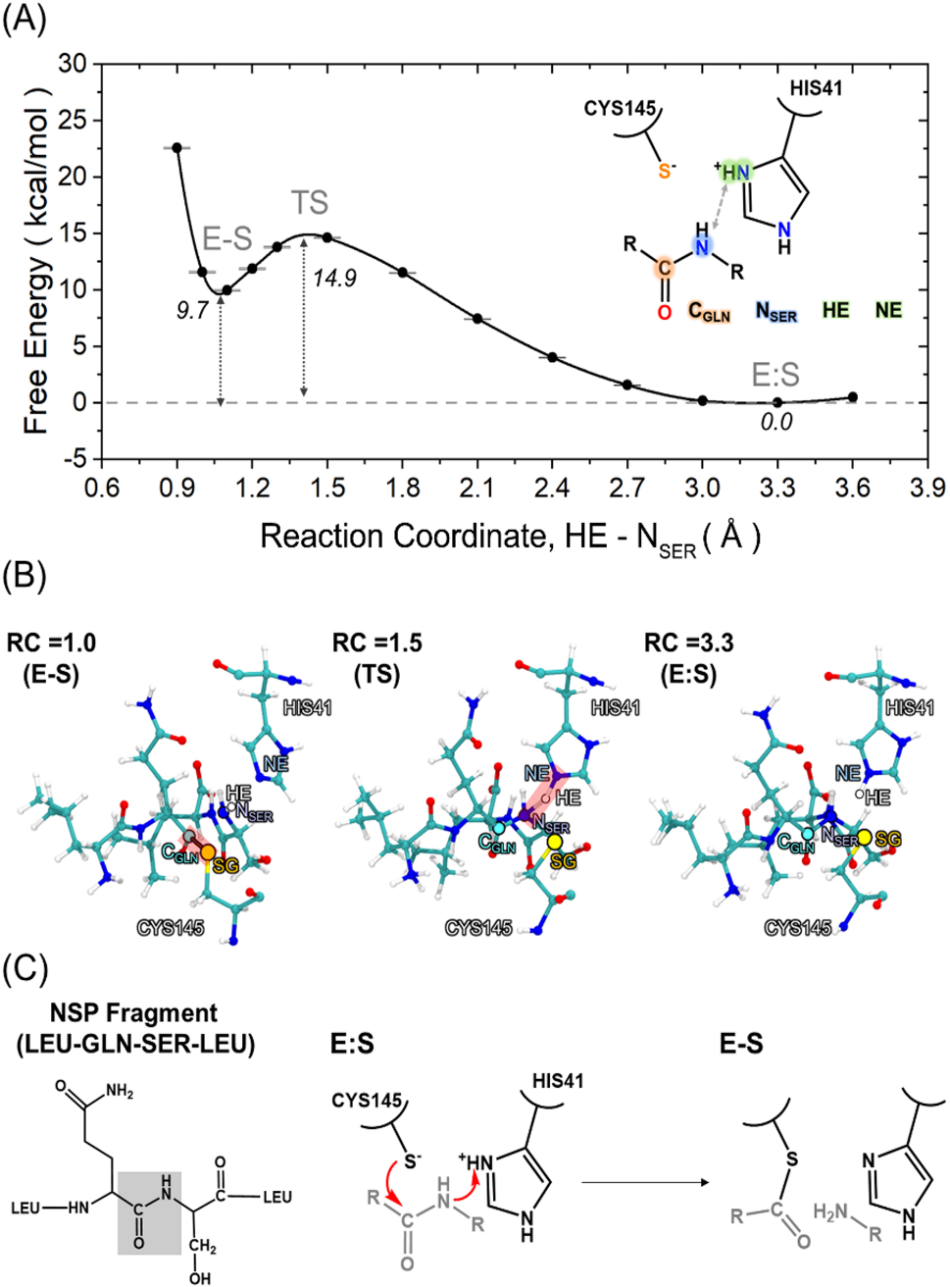
(**A**) Free energy profile for the formation of E-S complex with the NSP fragment as a function of the HE − N_SER_ distance. (**B**) Representative snapshots taken at three different points along the reaction: the E:S Michaelis complex (left), transition state (middle), and E-S intermediate (right). (**C**) A proposed concerted reaction mechanism from the E:S state to E-S complex of the NSP fragment.

Two possible reaction routes can be envisioned for the formation of the covalent enzyme–substrate (E-S) complex from the Michaelis complex (E:S): one is a stepwise reaction whereby the nucleophilic attack and the proton transfer occur sequentially, and the other is a concerted reaction whereby these two steps occur simultaneously. The study of these processes, keeping into account the full chemical complexity of the reaction environment (substrate, catalytic cleft, and second and outer coordination effects due to the protein matrix and the solvent), represents a challenge as the reaction coordinate is not known a priori. This forces one to employ approximated reaction coordinates expressed in terms of simple geometrical parameters, such as atom–atom distances, whose identification is often based on chemical intuition. While the free energy of a chemical step does not depend on this choice, the activation barrier might be appreciably affected (typically overestimated). With this limitation in mind, first we explored the stepwise route by choosing the distance between the sulfur atom of the CYS145 (SG) and a carbon atom of the carbonyl of glutamine (C_GLN_) in NSP as a reaction coordinate (Fig. S2). However, these simulations failed to identify the tetrahedral intermediate, which suggests that the stepwise mechanism is not energetically favorable (Fig. S2). Instead, simulations performed by choosing the distance between the hydrogen atom at the epsilon position of HIS41 (HE) and a nitrogen atom of the backbone of serine (N_SER_) of the substrate yields the formation of a thioester intermediate with the concomitant breaking of the peptide bond between GLN and SER (Figs. 5, S3). As the peptide N_SER_ is protonated by HIS41, the distance between S_γ_ and C_GLN_ becomes shorter, and the C_GLN_ − N_SER_ distance increases (Figs. 5B and S3). The free energy barrier for this concerted process is Δ*G*^‡^ = 14.9 ± 0.1 kcal/mol and with the resulting thioester at Δ*G*° = 9.7 ± 0.1 kcal/mol above the E:S complex. These values should be contrasted to those obtained from QM/MM calculations of the cleavage of *N*-methyl-acetamide by papain cysteine protease^33^, where the amide hydrolysis was found to follow a concerted mechanism with an activation free energy of 20.1 kcal/mol.

In summary, simulations suggest that M^PRO^ cleaves the NSP fragment with the concerted nucleophilic attack of CYS145 to the carbonyl group of the peptide bond, protonation of the NH group of the peptide bond, and breaking of the peptide bond.

### Reactions with the activated ester candidate

The proposed reaction path for the inhibition by the activated ester candidate follows that of hydrolysis of the NSP fragment. It starts with the deprotonation of the thiol group of CYS145 by HIS41 to promote its nucleophilic attack. Then a nucleophilic attack by CYS145 to the carbonyl carbon of the inhibitor is followed by the elimination of the methoxy group with the concomitant proton transfer from HIS41 to the anionic oxygen to form a thioester enzyme-inhibitor (E-I) complex and methanol as the leaving group.

We explored both the stepwise and concerted routes. The stepwise reaction was studied by using the distance between S_γ_ (SG) and a carbonyl carbon atom of an activated ester inhibitor candidate (C_AE_) as a reaction coordinate. However, like the reaction with the NSP fragment, no favorable formation of the covalent adduct was found.

**Figure 6:**
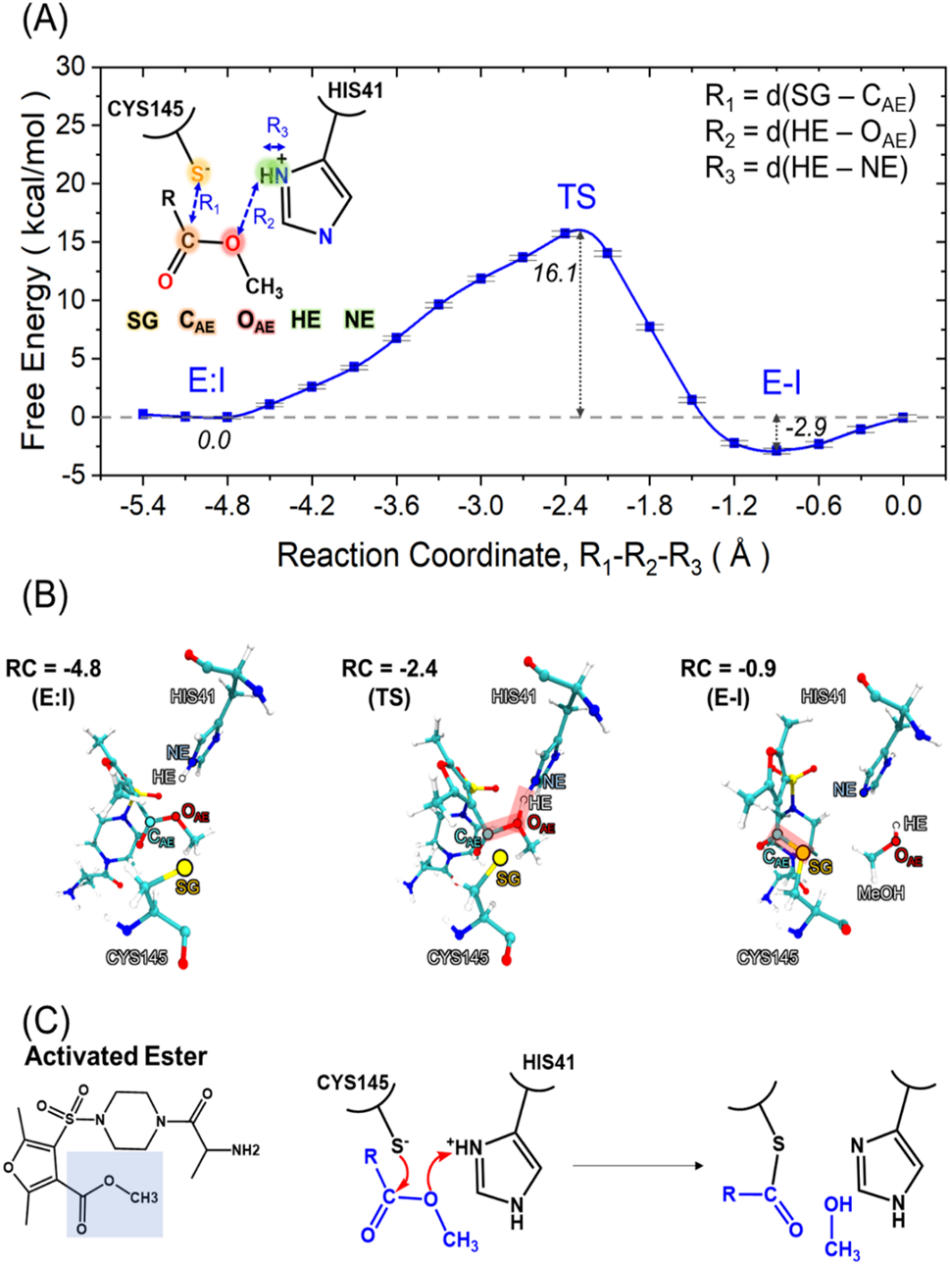
(**A**) Free energy profile of the activated ester inhibitor along the reaction coordinate, R_1_—R_2_—R_3._ (**B**) Representative snapshots taken at three different points along the reaction: E:I (left), transition state (middle), and E-I state (right). (**C**) A proposed concerted reaction mechanism from the E:I state to E-I complex of the activated ester inhibitor.

The concerted process was first explored by protonating an oxygen atom of AE (O_AE_). We used the HIS41 (HE)—O_AE_ distance as the reaction coordinate. However, these simulations did not lead to the formation of the E-I complex. This is likely because the formation of the S_γ_–C_AE_ bond and the breaking of the ester C_AE_–O bond is slow and cannot be observed in the time scale of our simulations, in contrast to the similar process in the NSP fragment whereby the breaking of the peptide bond is nearly spontaneous upon protonation of the backbone N by HIS41. To capture the simultaneous formation of the S_γ_–C_AE_ bond and the breaking of the ester C_AE_–O_AE_ bond, we resorted to the linear combination of distances R_1_–R_2_–R_3_ as the reaction coordinate, where R_1_ is the distance between SG and C_AE_, R_2_ is the distance between the HE and the oxygen of the methoxy moiety of the inhibitor (O_AE_), and R_3_ is the distance between HE and a nitrogen atom at the epsilon position of HIS41 (NE). A similar distance function was used as the reaction coordinate to compute the free energy profiles of heteroaryl nitrile inhibitors reacting with another cysteine protease, Rhodesain^34^. Using this reaction coordinate, the formation of the E-I complex and cleavage of the methoxy group from the activated ester inhibitor occurs in a concerted manner with an activation barrier of Δ*G*^‡^ = 16.1 ± 0.2 kcal/mol, Fig. 6). As the E-I complex is formed, the S_γ_ − C_AE_ and H_ε_ − O_AE_ distances decrease while the N_ε_ − H_ε_ and C_AE_ − O_AE_ distances increase (Fig. S3). Unlike the case with a NSP fragment, the E-I complex is energetically more stable than the E:I state (Δ*G*° = − 2.9 ± 0.2 kcal/mol, Fig. 6). This is clearly a prerequisite for inhibition.

### Reactions with the acrylamide candidate

Inhibition by the acrylamide candidate differs from that of NSP fragment and the activated ester inhibitor because the chemical nature of the acrylamide precludes the elimination step (i.e., the elimination of the amino fragment in NSP or methanol in the activated ester). The nucleophilic attack of the thiolate yields an enolate intermediate, which is then protonated by HIS41 leading to the formation of a *β*-thioether.

**Figure 7:**
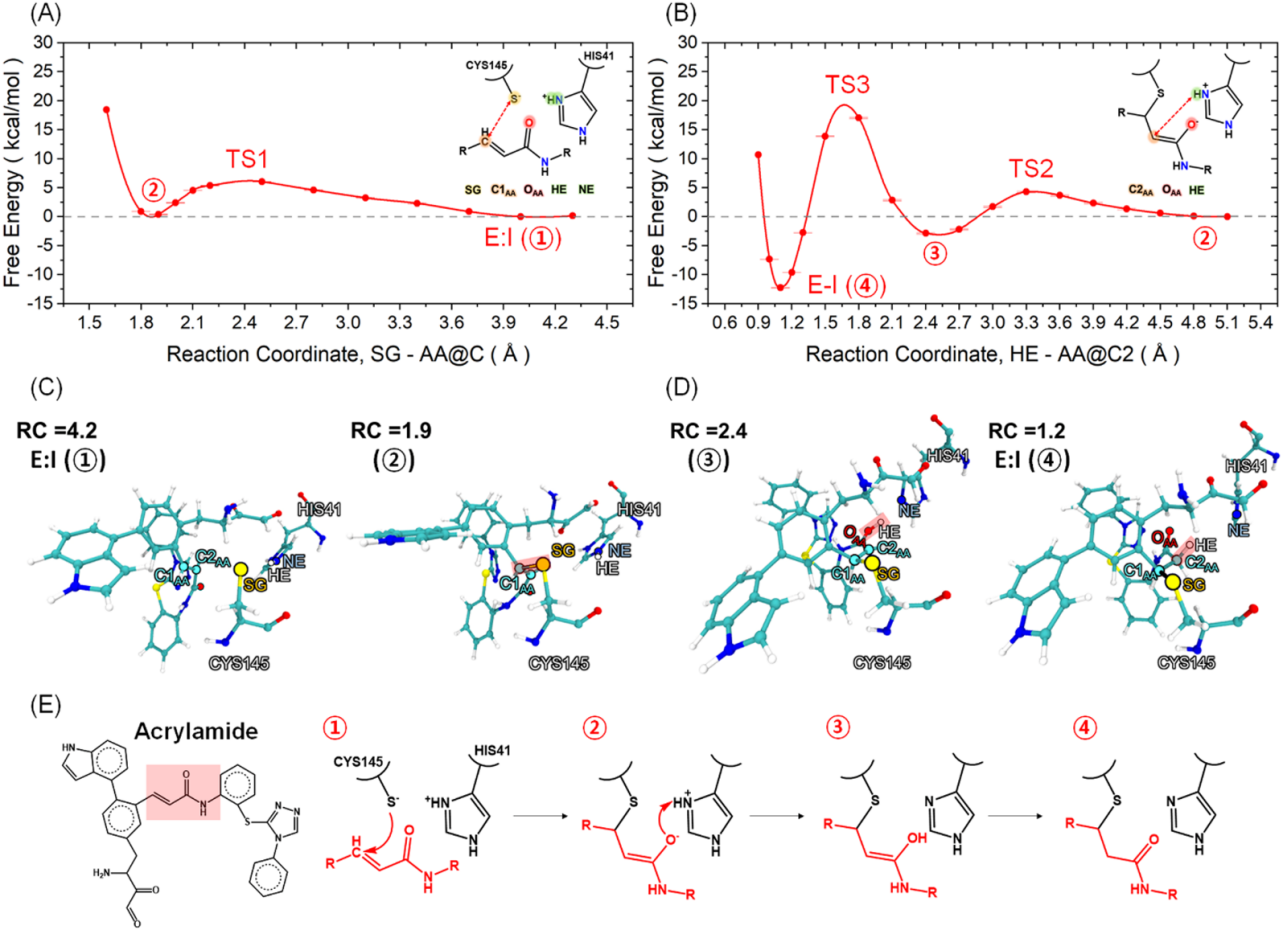
Free energy profile of the acrylamide inhibitor along the reaction coordinate, (**A**) distance between a SG atom of CYS145 and a C1_AA_ atom of acrylamide and (**B**) distance between a HE atom of HIS41 and C2_AA_ atom of acrylamide inhibitor_._ Representative snapshots were taken at different points of the reaction coordinate: highlighting (**C**) E:I formation (①), and the tetrahedral intermediate (②); (**D**) Second intermediate with a protonated oxygen atom (③), E-I complex (④) of the acrylamide inhibitor in M^PRO^. A proposed stepwise reaction mechanism from the E:I state to E-I complex of the acrylamide inhibitor.

Our simulations show that acrylamide forms a stable tetrahedral intermediate, unlike the NSP fragment and the activated ester. We performed two separate QM/MM constrained MD simulations to explore both the stepwise and concerted routes for the nucleophilic attack and protonation of the enolate. As for the stepwise route, we explored the reaction path between the E:I state and the covalent complex E-I (structures 1 and 2 in Fig. 7A) using the SG − C1_AA_ distance. This was followed by additional free energy calculations using HE − C2_AA_ distance for the free energetics of protonation and conversion of the enol to the ketone (structures 2, 3, and 4 in Fig. 7B). Formation of the enolate (structure 2) from E:I is equiergic (Δ*G*° = 0.0 ± 0.1 kcal/mol) and requires the crossing of a small activation barrier Δ*G*^‡^ = 6.3 ± 0.1 kcal/mol; then, a proton transfer from HIS41 to the enolate oxygen atom (Δ*G*^‡^ = 4.3 ± 0.2 kcal/mol) leads to the formation of enol intermediate (structure 3, Δ*G*° = − 3.1 ± 0.1); and isomerization to the keto form (structure 4) as the *β*-thioether requires an activation energy barrier of Δ*G*^‡^ = 19.3 ± 0.1 kcal/mol (Figs. 7 and S3). Formation of the E-I from E:I is favorable by Δ*G*° = − 12.2 ± 0.1 kcal/mol. Surprisingly, proton transfer from HIS41 to O_AA_ and the subsequent keto-enol equilibrium were not considered for previously suggested acrylamide inhibitors^16^.

As for the concerted process, the chemical nature of the enzyme-AA covalent complex precludes the use of the same reaction coordinates adopted for the NSP fragment and the AE. Instead, in this case, we used the HE − C2_AA_ distance as the reaction coordinate. By doing so, the formation E-I complex is achieved after the crossing of a barrier Δ*G*^‡^ = 28.5 ± 0.5 kcal/mol. This barrier is considerably higher than that of the stepwise process (19.3 ± 0.1 kcal/mol) and therefore the concerted process should be considered kinetically disfavored over the stepwise process. The free energy change of the E-I complex along the concerted route is Δ*G*° = − 10.4 ± 0.5 kcal/mol, which is consistent with that of the stepwise route, -12.2 ± 0.1 kcal/mol (Fig. S4). The small difference between the two values should be taken as a measure of the statistical error and the bias introduced by the two different choices for the reaction coordinate.

In summary, both the activated ester and the acrylamide form stable E-I intermediates (Δ*G*° = − 2.7 kcal/mol and − 12.2 kcal/mol, respectively). The stability of the E-I intermediate is a prerequisite for inhibitory activity. In contrast, the formation of the thioester intermediate formed from the NSP fragment (+ 9.7 kcal/mol) is unfavorable.

### Hydrolysis of the thioester intermediate

Proceeding forward in the hydrolysis of the NSP substrate, the C–S bond in the E-I complex is hydrolytically cleaved by water, the fragment of the product with a carboxylic end is released and the CYS145 is regenerated. The intermediate from the activated ester substrate could be also hydrolytically released. Instead, the β-thioether bond formed by the acrylamide is not easily hydrolyzable. It is known that thioethers have higher stability against many aggressive conditions, such as treatments with organometallic reagents, when compared to thioesters or disulfides^35^.

There are several water molecules adjacent to the C–S bond of the E-S (NSP) or E-I (activated ester) complex that can carry out hydrolysis. The hydrolysis mechanism was not explicitly investigated. Rather the free energy for the hydrolysis of E-I from NSP and the activated ester was estimated from truncated structural models extracted from the QM/MM simulations (Fig. S5). As shown in Fig. S5 and Table S1, hydrolysis is slightly endoergic for NSP (Δ*G*° = 1.5 kcal/mol) and rather endoergic for the activated ester (Δ*G*° = 6.9 kcal/mol). The free energy for NSP is consistent with the previous QM/MM studies of the hydrolysis of the C–S in cruzain cysteine proteases (Δ*G*° = 0.8 kcal/mol)^36^.

## Conclusions

Accurate identification of hits and lead optimization are crucial for the development of novel therapeutics to combat the threat posed by current and emerging viruses. Therapeutic candidates interact with target proteins either by forming a covalent bond or non-covalently through non-bonding interactions^37–41^. A significant need exists for the development of small-molecule inhibitors that directly target proteases^42^. We performed a high throughput virtual screening of potential covalent inhibitors of the SARS-CoV-2 M^PRO^. The screening workflow employed an extensive dataset of antiviral compounds^28,29^ to train a 3D-Scaffold deep learning model to generate new antiviral candidates^17^. We found that compounds with acrylamide^30^ and ester warheads^31^ show promise to be consider as a covalent inhibitor of M^PRO^. The potential inhibitory activity of two top-ranking acrylamide and ester warheads was further investigated via classical and hybrid QM/MM MD simulations. The mechanism of hydrolysis of a fragment of a non-structural protein was also studied for reference.

**Figure 8:**
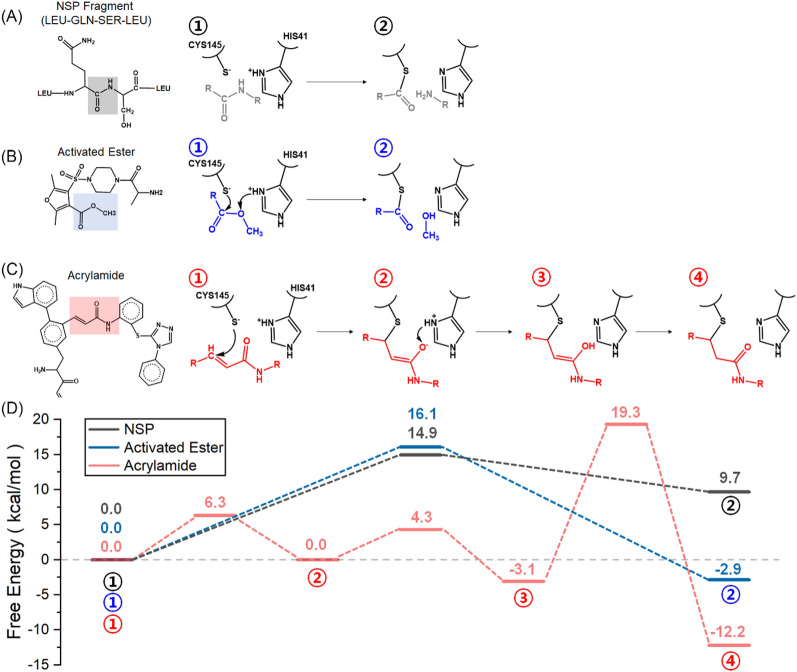
Proposed reaction mechanisms of (**A**) NSP fragment, (**B**) activated ester, and (**C**) acrylamide inhibitor candidates based on the simulations. (**D**) Free energy profiles from the free enzyme with the substrate (E:S state for the NSP) or with the inhibitors (E:I) to the enzyme–substrate (E-S) or enzyme-inhibitor (E-I) complex of three substrates used in this study, which are relative to the energy of E:I state (①).

Classical MD simulations revealed that the presence of the NSP fragment and the inhibitor candidates in the active site does not affect the global dynamics of M^PRO^. However, two regions near the active site undergo significant structural changes upon substrate binding. The energetics for covalent binding of the chosen two warheads to CYS145 of M^PRO^ was explored by QM/MM simulations and compared with that of NSP. The overall sequence of reactions for the candidate inhibitors and NSP are reported in Fig. 8. The simulations suggest a concerted nucleophilic attack of the deprotonated CYS145 to the carbonyl group of NSP or the activated ester with the protonation of the peptide nitrogen (NSP) or the oxygen atom of the activated ester by HIS41 and the concomitant dissociation or an amino terminated fragment (NSP) or methanol (activated ester). In contrast, acrylamide reacts with M^PRO^ in a stepwise manner. It is found that both activated ester and acrylamide form stable covalent complexes in contrast to the thioester formed from the NSP. This is a clear requirement for efficient covalent inhibitors. Also, inhibition by the activated ester could be reversible as the resulting thioester can be hydrolyzed to regenerate the free enzyme, whereas inhibition by acrylamide is instead expected to be irreversible. Taken all together, these results highlight that both activated ester and acrylamide-based candidates can serve as inhibitors of M^PRO^.

## Methods

### Covalent inhibitor design

We recently developed the Automated Modeling Engine for Covalent Docking using AutoDockFR docking^43^. This pipeline contains all the pieces needed to automate the covalent docking of specified electrophilic warheads to a receptor CYS sidechain of the protein target. Given a protein target and a list of SMILES, the pipeline forms a covalent bond between the thiol of the CYS sidechain and the pertinent functional group(s) of a ligand, and then determines the energetically favorable poses of the ligand. Our workflow utilizes RDKit^44^ and reaction SMART to prepare, predict, and rank binding poses for a set of input molecules. The automated workflow takes a prepared receptor, which defines the binding site, and a list of ligands in SMILES format. The SMILES of each ligand/compound is searched for substructures with defined thiol addition reactions and produces molecules in PDB format with an appropriately attached cysteine sidechain. The prepared ligand is then passed to AutodockFR, which places the ligand at the binding site and searches the conformational space. AutodockFR places the ligand by superimposing the added cysteine sidechain with the equivalent atoms of the receptor and utilizes a genetic algorithm to explore favorable binding sites and conformations.

Scores are defined in terms of various ligand-ligand and ligand–protein interaction forces, including van der Waals, electrostatic, hydrogen bonds, and desolvation contributions. We present the absolute values of the docking score (Fig. 2), whereby a higher score implies tight binding between the ligand and receptor. For each molecule, all individually calculated pose values are averaged. The receptor was kept rigid during the docking.

### MD simulations

Simulations were started from a recently reported crystal structure of SARS-CoV-2 3CL M^PRO^ (PDB ID: 6WQF)^11^. MD simulations of dimeric M^PRO^ with no substrates (apo-enzyme), with NSP fragment, and the two inhibitor candidates were performed. The simulations with substrates bound to M^PRO^ were prepared by binding and docking either the candidate inhibitors or a NSP fragment using Discovery Studio Visualizer 19^45^ to the cysteine at position 145. The initial poses for covalent inhibitor candidates and covalently bonded NSP fragment were taken from the poses with the highest scores after the docking. In fact, the exploration of the conformational space of a protein/ligand complex where a pose of the ligand is not known a priori is challenging. To cope with this issue, MD simulations were started from the most favorable pose of the ligands as obtained from the virtual high-throughput covalent screening process described in the “Covalent Inhibitor Design” Section. Hydrogen atoms were added using pdb2gmx tools in GROMACS 2018.6 package^46^ and PROPKA 3^47,48^ was used to calculate the protonation states of amino acids at pH 7.0. We adopted the Amber FF14SB force field^49^ for the M^PRO^ and the NSP fragment along with the general AMBER force field (GAFF)^50^ for the covalent inhibitor candidates including the activated ester and acrylamide inhibitors. Partial charges of the covalent inhibitors including CYS145 were obtained based on the restrained electrostatic potential atomic partial charge (RESP) method^51^ at HF/6-31G* level using NWChem 6.8.1^52^. The TIP3P water model^53^ was used along with the Joung and Cheatham ion parameters^54^ for the neutralizing monovalent ions. The initial system was relaxed by the conjugated gradient algorithm up to a maximum residual force of 10.0 kJ/mol nm^2^. Then, the system was equilibrated at 300 K for 500 ps under NVT ensemble using the Berendsen velocity rescaling method followed by 500 ps with NPT ensemble using the Berendsen pressure coupling method^55^. Harmonic restraints with the force constant of 1000 kJ/mol·nm^2^ were applied on the protein and substrate during equilibration steps. After the equilibration, we performed production runs for 100 ns at 300 K and 1 atm using the Parrinello-Rahman pressure coupling method^56^ while all the restraints were released at this step. All simulations were performed with a timestep of 2 fs. The particle mesh Ewald algorithm^57^ was used to evaluate long-range electrostatic interactions. GROMACS 2018.6^46^ was used for all-atom classical MD simulations.

### Constrained QM/MM MD simulations

The reaction of M^PRO^ with NSP and the candidate inhibitors was studied using hybrid QM/MM molecular dynamics simulations in the NVT ensemble with the Nose–Hoover thermostat^58–60^. In these simulations, CYS145, doubly protonated HIS41, and covalent inhibitors or NSP were described using density functional theory while the rest of the system using the same empirical force field adopted in the classical MD simulations. The PBE exchange and correlation functional augmented with Grimmes’ dispersion corrections^61^ in conjunction with the triple-zeta TZVP basis set^62^ was used to describe the valence electrons and Goedecker-Teter-Hutter (GTH) pseudopotentials^63^. Previous works reported in the literature demonstrated that the PBE functional well captures interactions important for the recognition of the natural M^PRO^ substrates^64^ or inhibitors^65^. Free energy profiles were obtained from constrained MD simulations by integrating the potential of mean force obtained as the ensemble average of the Lagrangian multipliers^66^ of the imposed constraints.

Different reaction coordinates expressed in terms of distances were explored as described in the text. Reaction coordinates were sampled at increments of 0.3 Å. Statistical errors on free energies were obtained by estimating the uncertainty on the ensemble averages via block averages and from standard error propagation formulas. These QM/MM simulations were started from configurations taken at the last frame of the corresponding classical MD simulation. All QM/MM simulations were performed with the code CP2K 7.1.0^67^ using an integration timestep of 0.5 fs.

### Analyses

Analyses used in this study such as root means square deviations (RMSD), atomic fluctuations (RMSF), distance analyses, etc., were carried out using the analysis tools in GROMACS^46^, CPPTRAJ in Ambertools package^68,69^, or Plumed 2.4.0^70,71^. VMD 1.9.3^72^ was used for visualizing simulation trajectories as well as taking and rendering the snapshots of simulations.

## Associated content

### Supporting information

Root means square deviations and fluctuations of a NSP fragment, and two inhibitors bound to the M^PRO^; free energy profiles suggesting that the formation of M^PRO^—NSP complex does not follow a stepwise mechanism; free energy differences between E-S (or E-I) state and E:P state of the NSP fragment and activated ester inhibitor.

## Supplementary Information

Supplementary Information.

## Data Availability

The datasets for the docking simulations and results used and/or analysed during the current study available in this published article and in the GitHub repository, https://github.com/nkkchem/AMECovDock/tree/main/data. The datasets for the results of MD simulations used and/or analysed during the current study available in this published article and its supplementary information files.
