# Supplementary material for: Mechanistic investigation of SARS-CoV-2 main protease to accelerate design of covalent inhibitors
Source: Sci Rep. 2022 Dec 5;12:21037. doi: 10.1038/s41598-022-23570-6 (PMC9722715; doi:10.1038/s41598-022-23570-6)
Supplement: Supplementary file 1 — Supplementary Information. [file 41598_2022_23570_MOESM1_ESM.docx]

Mechanistic Investigation of Main Protease SARS-CoV2 to Accelerate Covalent Inhibitors Design

Hoshin Kim^1^, Darin Hauner^2^, Joe Laureanti^1^, Kruel Agustin^2^, Simone Raugei*^1^, Neeraj Kumar*^2^

^1^Physical and Computational Science Directorate, Pacific Northwest National Laboratory, Richland, Washington 99352, United States,

^2^Earth and Biological Science Directorate, Pacific Northwest National Laboratory, Richland, Washington 99352, United States,

**Figure S1**. (A) Root mean square deviations (RMSD) and (B) root mean square fluctuations (RMSF) of a NSP fragment (black), the activated ester inhibitor (red), and the acrylamide inhibitor (blue). (C) Root mean square fluctuations (RMSF) observed from MD of the M^PRO^ subunit with respect to the covalently bound hits (nonstructural protein (NSP), ester, and acrylamide warhead). The NSP fragment (black), activated ester (blue), and acrylamide inhibitor (red) are covalently bound to one subunit while no substrates are placed in the other one. The same RMSF calculations are done for the apo-enzyme (thick translucent line). (D) Snapshots highlighting three regions where relatively high fluctuations are observed in all four cases.


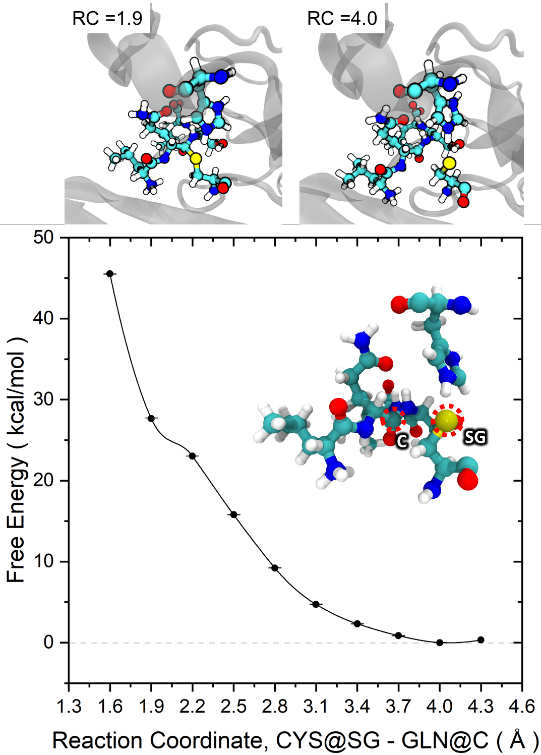


**Figure S2.** Free energy profile along the reaction coordinate SG – C_AE_ distance (bottom) and representative snapshots taken at RC = 1.9 (top left) and 4.0 (top right). This free energy profile suggests that NSP does not follow stepwise mechanisms


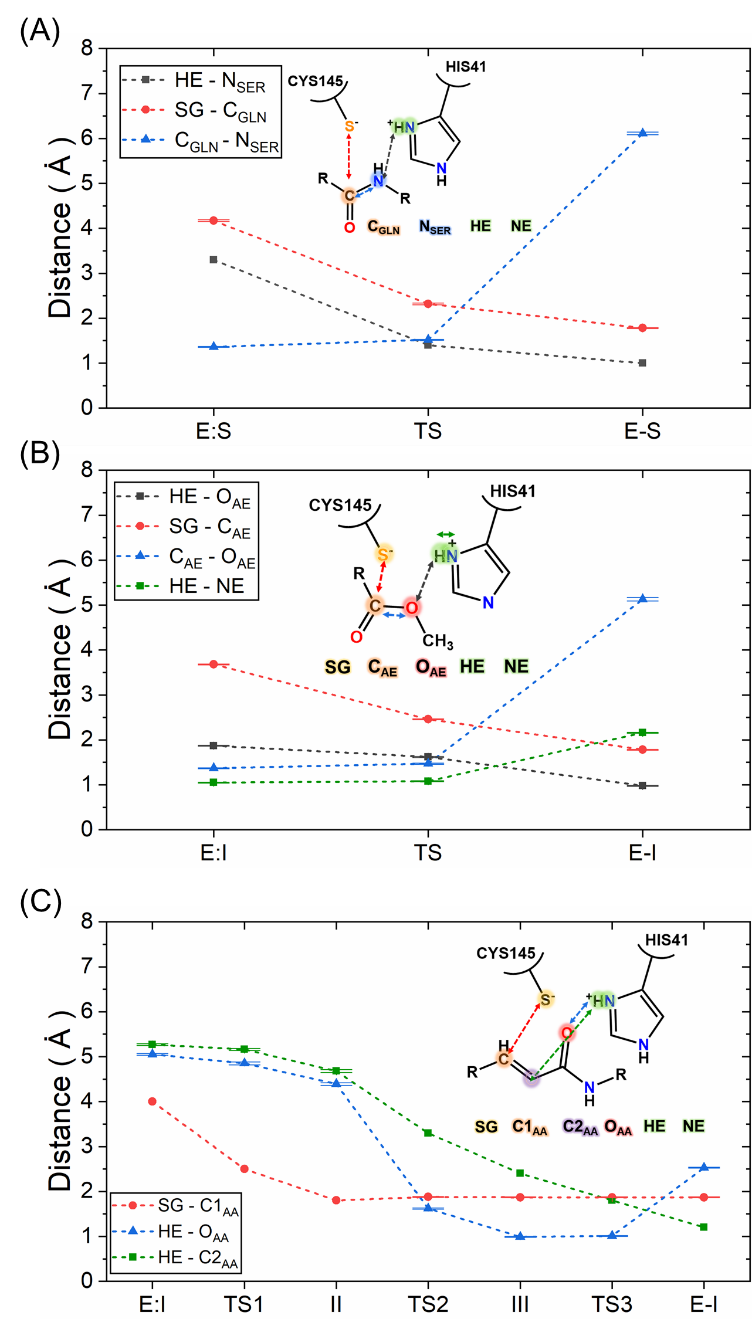


**Figure S3.** (A) H_ε_ (HE) – N_SER_ (black), S_γ_ (SG) – C_GLN_ (red), and C_GLN_ – N_SER_ (blue) distance along the reaction path of NSP fragment. (B) HE – O_AE_ (black), SG – C_AE_ (red), C_AE_ – O_AE_ (blue), and HE – N_ε_ (NE) distances along the reaction path of the activated ester candidate. (C) SG – C1_AA_ (red), HE – O_AA_ (blue), and HE – C2_AA_ (green) distances along the reaction path of the acrylamide candidate. Average distances are calculated by simulation data with several different reaction coordinates representing the E:S or E:I, TS, and E-S or E-I. Standard deviations are given in the parentheses.


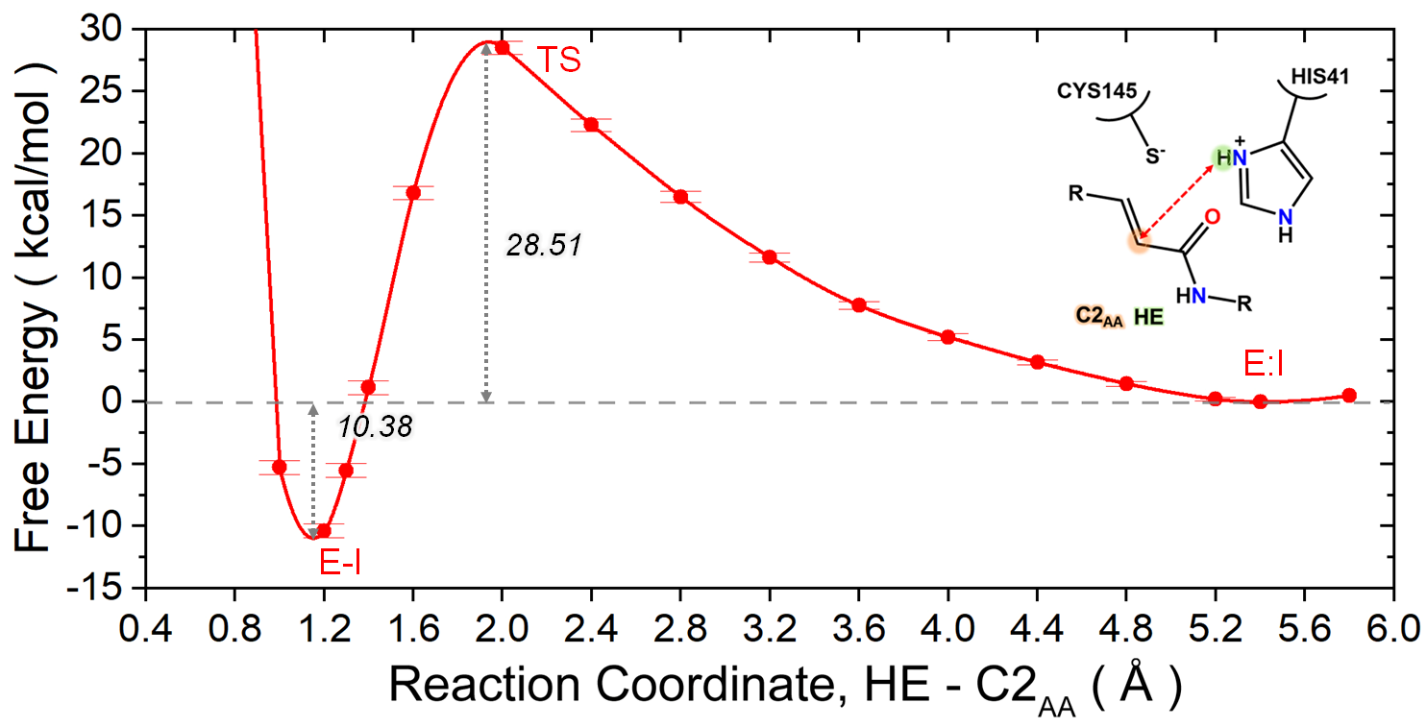


**Figure S4.** Free energy profile along the reaction coordinate, HE – C2_AA_ distance. This free energy profile suggests that acrylamide inhibitor can possibly react with M^PRO^ in a concerted manner.


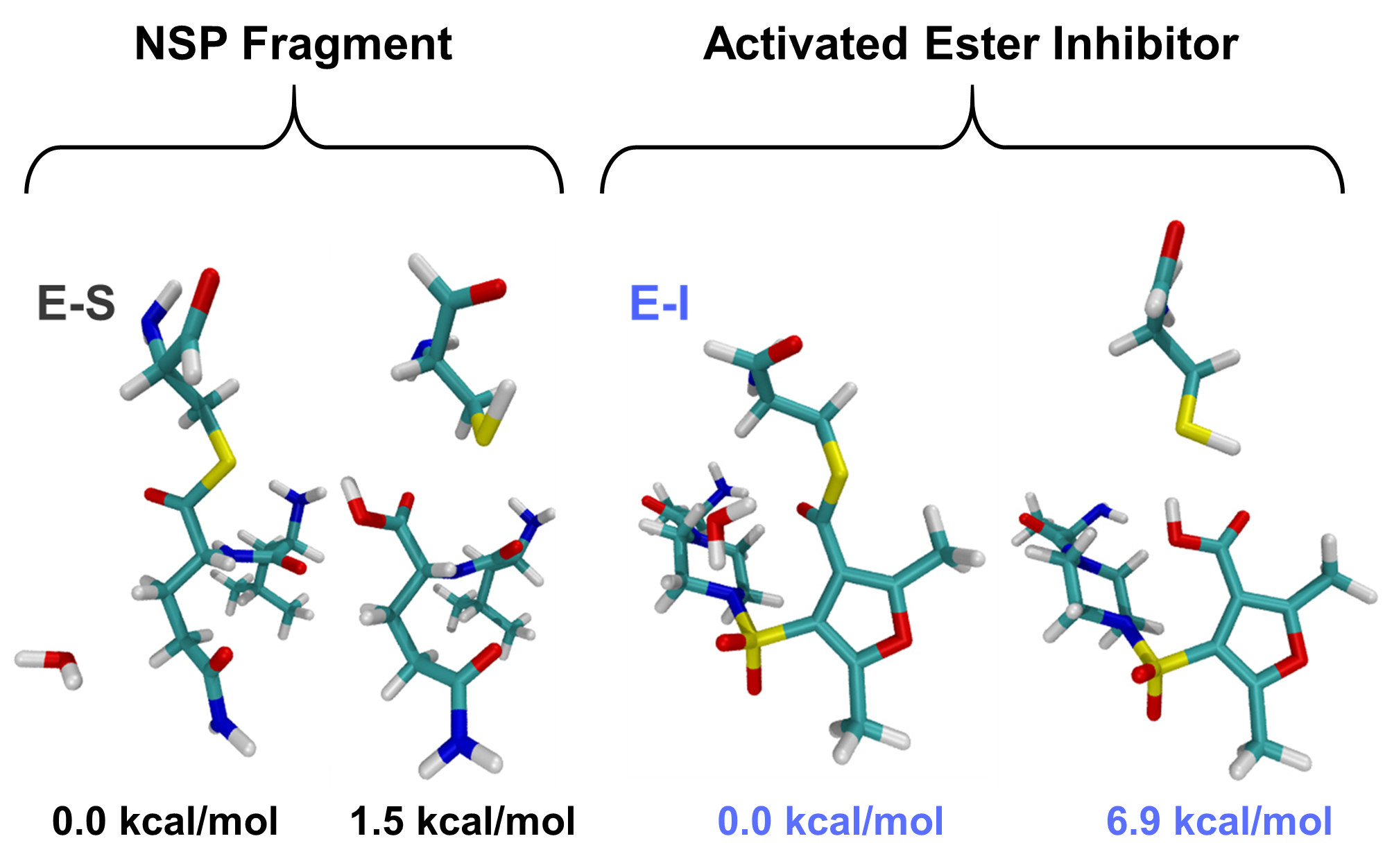


**Figure S5.** Structural models used for the calculation of free energy of hydrolysis of the C - S bond in E-S (NSP) and E-I (activated ester) to regenerate CYS145 and form the product with a carboxylic end.

**Table S1**. Electronic and free energies of the E-S / E-I and the E:P state of the NSP fragment and the activated ester inhibitor. The PBE functional with TZVP-GTH-MOLOPT basis set was used for these calculations. EE is electronic energy. Gibbs free energy is calculated by EE + H_corr_ - TS where H_corr_ is a thermal correction to enthalpy and TS is total entropy. ∆G is relative free energy relative to the E-S or E-I state.

|  |  | EE  (Hartree) | H_corr_  (Hartree) | TS  (Hartree) | ∆G  (kcal/mol) |
| --- | --- | --- | --- | --- | --- |
| NSP | E-S | -226.5045 | 0.3780 | 0.0737 | 0.0000 |
|  | E:P | -226.5017 | 0.3778 | 0.0739 | 1.4757 |
| Activated  Ester | E-I | -285.7629 | 0.4138 | 0.0786 | 0.0000 |
|  | E:P | -285.7493 | 0.4120 | 0.0794 | 6.9381 |
